# Beneficial Roles of Microglia and Growth Factors in MS, a Brief Review

**DOI:** 10.3389/fncel.2020.00284

**Published:** 2020-09-23

**Authors:** Vincent Pons, Serge Rivest

**Affiliations:** Neuroscience Laboratory, Department of Molecular Medicine, Faculty of Medicine, CHU de Québec Research Center, Laval University, Québec City, QC, Canada

**Keywords:** microglia 1, growth factors, remyelination, multiplesclerosis, innate immnuity

## Abstract

Microglia are the brain resident immune cells; they can produce a large variety of growth factors (GFs) to prevent neuronal damages and promote recovery. In neurodegenerative diseases, microglia can play both benefic and deleterious roles, depending on different factors and disease context. In multiple sclerosis, microglia are involved in both demyelination (DM) and remyelination (RM) processes. Recent studies suggest a beneficial role of microglia in regenerative processes. These include the regenerative development of myelin after DM. This review gives an overlook of how microglia and GFs can influence the RM properties.

## Introduction

Microglia belong to the immune system. There is a general consent on the involvement of microglia in neurodegenerative diseases. In multiple sclerosis (MS), microglial cells may have both detrimental and beneficial actions, depending on the circumstances, models, and disease progression and severity. Tissue examination as well as experimental findings show that demyelination (DM) is usually associated with a robust inflammation and the presence of immune cells (Kotter et al., 2005). Microglia are involved in each MS phase; they are one of the main cell types responsible for phagocytic clearance of myelin debris and are able to release a wide range of cytokines and growth factors (GFs) known to contribute to the remyelination (RM; Hinks and Franklin, 1999; Barnett and Prineas, 2004; Glezer et al., 2007; Rivest, 2009). RM is the regenerative process following natural or induced DM occurring in the adult central nervous system (CNS). RM is composed of two major phases. The first consists of the colonization of lesions by oligodendrocyte progenitor cells (OPCs), and the second consists of the differentiation of OPC into mature oligodendrocytes (ODs), which will generate functional myelin sheaths (Chari, 2007). OPCs and their mature counterpart are partially driven by microglia-secreted cytokines and GFs.

This brief review is an overview of the involvement of microglia in RM process through the production of specific GFs.

## Microglia

Microglia are the innate immune cells of CNS. They arise from the yolk sac and invade the newly formed brain in mice between embryonic days 8.5 and 10. These monocytic lineage-derived cells maintain themselves in the brain *via* self-renewal (Rossi and Lewis, 2018). Microglia represent 5% to 15% of adult brain cells (Thion et al., 2018).

They continuously survey the CNS with their motile processes and are the first responder to insults, such as pathogen infections, toxins, and brain injuries (Shemer et al., 2015; Li and Barres, 2018). Microglia are also known to contribute to CNS development and homeostasis (Li and Barres, 2018; Thion et al., 2018) and are involved in neurogenesis and synaptic pruning and also participate to neuronal support and myelogenesis (Nagata, 1997; Paolicelli et al., 2011; Lampron et al., 2015; Shemer et al., 2015). Microglia are highly plastic. They can be polarized and can produce and release a large variety of mediators ranging from cytotoxic such as interleukin 1β (IL-1β), tumor necrosis factor α (TNF-α), reactive oxygen species, and nitric oxide (NO), IL-4, to trophic factors that provide support to CNS, notably with brain neurotrophic–derived factor (BDNF), insulin like growth factor 1 (IGF-1), arginase-1 (Arg-1), and transforming growth factor β (TGF-β; Ueno et al., 2013). BDNF is a neurotrophic factor, acting on neurons. It supports survival and promotes the growth and differentiation of newly formed neurons and synapses (Flores et al., 2020). IGF-1 inhibits apoptosis and promotes proliferation and differentiation of neural stem cells (Thored et al., 2009). Arg-1 outcompetes inducible NO synthetase, decreasing the level of NO, thus the inflammation. TGF-β acts on inflammation and microglial proliferation. It suppresses the expression of MHC-II, IL-1, and TNF-α (Lee et al., 2017).

The role of microglia under pathologic conditions has been highly studied, and they are believed to be a key therapeutic target in CNS diseases.

## A Global Description of MS

MS is an autoimmune and neurodegenerative disease that affects the CNS. The pathology appears between the ages of 20 and 40 years with an autoimmune reaction against components of myelin (Huang et al., 2017). MS affects sensation and motor, autonomic, and neurocognitive function (Sospedra and Martin, 2016). The relapsing–remitting (RR) form is the most common subtype of MS. This form is characterized by acute demyelinating phase, corresponding to an attack on myelin and nerve fibers by immune cells, followed by periods of remission (Ghasemi et al., 2017). Usually, it lasts 5–10 years and evolves in 80% of cases in secondary chronic–progressive phase (Nazareth et al., 2018). The neuroinflammation and by extension damages in the CNS are due to immune cell activation and their cytokines, resulting in the formation of plaques composed of cells, demyelinated axons, and astrogliosis in the white and gray matter (Compston and Coles, 2008). The inflammation in MS is dominated by T cells and macrophages, but MS is mainly thought to be a T cell–mediated autoimmune disease. Antigen-presenting cells following toll-like receptor stimulation produce cytokines, which shape the differentiation of T cells into a specific subsets (Legroux and Arbour, 2015). CD8^+^ T cells seem to be the dominant population of T cells (Rangachari et al., 2017), because CD4^+^ T cells do not exceed 20% to 30% of the total T-cell population (Kutzelnigg and Lassmann, 2014). Based on secretion of specific molecules, three main subtypes of T helper (T_H_) cells have been described; T_H_1 (interferon- γ), T_H_2 (IL-4, IL-5, and IL-13), and T_H_17 (IL-17, IL-21, and IL-22). Noteworthy, T_H_17 phenotype favors the recruitment of neutrophils and the activation of innate immunity (Raphael et al., 2015). In MS lesion, CD4^+^ and CD8^+^ express IL-17. CD4^+^ T_H_17 are necessary to develop autoimmune encephalomyelitis (EAE), the main animal model MS (Komiyama et al., 2006). Macrophages and activated microglia are in contact with myelin sheaths, as well as dystrophic axons, and have the ability to phagocyte myelin debris and produce proinflammatory molecules during DM (Brück et al., 1995; Hänninen, 2017).

However, the role of adaptive immunity may not be necessarily harmful. Some studies reveal that neurotrophins such as BDNF are synthesized in active MS plaques (Kutzelnigg and Lassmann, 2014). Moreover, T_H_2 promotes anti-inflammatory response (Ghasemi et al., 2017; Mahallawi et al., 2018).

RM is spontaneous; myelin sheaths are newly formed around axons in CNS following DM. However, RM becomes incomplete or fails with time. This newly formed myelin sheath differs from the original, as it is thinner and shorter (Goldschmidt et al., 2009; Cunniffe and Coles, 2019). The immune system is thought to be harmful in MS, but it is also essential for a proper RM. Indeed, RM is stimulated by the inflammatory process occurring at the DM phase. Myelin debris has an inhibitory effect for OPC differentiation, so its clearance is an important step for RM (Robinson and Miller, 1999; Kotter, 2006; Yong and Rivest, 2009; Cunniffe and Coles, 2019). Chari et al. (2006) have shown that anti-inflammatory molecules can inhibit differentiation of OPCs and delay RM. Other studies have demonstrated a less efficient RM in the absence of MHC-II, inflammatory factors, and active immune system (Arnett et al., 2003). Microglia are deeply involved in MS progression; these cells not only modulate OPC homeostasis, differentiation, maturation, and neurogenesis but also contribute to OD and neuronal death (Pang et al., 2010; Hagemeyer et al., 2017).

## Role of Microglia in RM

In MS and its animal models, microglia are highly activated and participate in all phases of the disease. Microglial cells show increased expression of inflammatory cytokines and cytotoxic molecules. An accumulating body of evidence supports rather a detrimental role of microglia in neurodegenerative diseases. However, numerous articles indicate that microglia could also exert a protective function in MS and drive the RM process (Lloyd and Miron, 2019). Inefficient microglial activation, phagocytosis, and inflammatory response toward myelin debris during DM process lead to an impaired remyelinating process (Neumann et al., 2008; Lampron et al., 2015; Pons et al., 2020). A change in microglia polarization from activation to repair profile can initiate the RM (Miron et al., 2013). The dual aspect of microglia has been shown *in vitro*. Lipopolysaccharide (LPS) treatment of microglia has deleterious consequences on neurons, whereas they become neuroprotective when preactivated with IL-4. Conditioned microglia downregulate the release of GFs by TNF-α (Butovsky et al., 2006). Production of GFs by microglia promotes survival and proregenerative activities on ODs and OPCs (Hsieh et al., 2004; Neumann et al., 2008; Voet et al., 2019). IGF-1 protects OPCs from TNF-α and enhances cell survival through PI3K/Akt pathway. Furthermore, IGF-1 signals suppress caspase-9 and caspase-3 activation, which protects OPCs from the mitochondrial apoptotic pathway (Pang et al., 2007). Concerning DM/RM, a reduced number and area of demyelinating lesions were found in EAE Lewis rats that received systemic IGF-1 injections. Moreover, these lesions contain a higher proportion of proliferative OD cells (Yao et al., 1995; Zeger et al., 2007). However, Cannella et al. (2000) used IGF-1 at different time points during acute and chronic phases of EAE in mice, and they failed to demonstrate a real efficacy of IGF-1 treatment. Their results reveal a transient clinical amelioration and a low RM level, which also did not improve in animals that received IGF-1 at chronic time points (Cannella et al., 2000; Zhang et al., 2011).

A marked increase in BDNF mRNA expression and a slight increase in neurotrophin-4/5 (NT-4/5) levels have been shown in LPS-treated microglia *in vitro*. These results demonstrate that inflammatory activated microglia have the ability to favor the secretion of GFs and possibly participate in neuronal regeneration and neuroprotection (Var and Byrd-Jacobs, 2020). The BDNF receptor is found at the site of MS lesion. Interestingly, BDNF is naturally reduced in corpus callosum after cuprizone treatment (VonDran et al., 2011). This neurotrophic factor is detected in macrophages/microglia and T cells in active and inactive lesions (Kerschensteiner et al., 1999; Kutzelnigg and Lassmann, 2014). Neurons are in distress during MS crisis, and a robust expression of BDNF receptors may compensate to balance the detrimental effects of inflammation (Stadelmann et al., 2002). Furthermore, neutralization of BDNF, NT-3, and nerve growth factor within the cultured tissues enhance MHC class II inducibility (Neumann et al., 1998; Rahimlou et al., 2020). Through this factor, microglia affect the inflammation by impairing the antigenic presentation and then promote the survival of existing neurons and promote neurogenesis and RM (Huang and Reichardt, 2001). BDNF impacts also ODs. Indeed, following cuprizone lesion in mice with a deficiency in BDNF production, VonDran et al. (2011) highlighted a reduced number of NG2-positive cells and deficit in myelin protein but no change in the total numbers of ODs. This suggests that BDNF may regulate the numbers of OPCs following DM. Interestingly, Fletcher et al. (2018) used a BDNF-like molecule, namely, TDP6, which mimics the TrkB-binding region of BDNF, and highlighted the beneficial effect of this molecule to promote myelin regeneration and OD differentiation in mice after a demyelinating insult.

Arginase (Arg-1) is an enzyme that converts arginine to ornithine. In multiple animal models, the internalization of myelin by microglia leads to the expression of Arg-1 (Miron et al., 2013; Guerrero and Sicotte, 2020). *In vitro*, both microglia and astrocytes produce arginase. Arg-1 activity can reduce the supply of arginine needed for the production of NO (Caldwell et al., 2018). In EAE model, Yang et al. (2016) used spermidine as a treatment, which abolished the expression of inflammatory factors while enhancing the expression of Arg-1 in immune cells and reverses EAE progression. Moreover, macrophages pretreated with Arg-1 inhibitor abrogate the therapeutic effect of spermidine. This study could suggest a partial role of Arg-1 in the disease progression (Yang et al., 2016), but the role of Arg-1 remains controversial. Arg-1 gene is highly up-regulated in the spinal cord of EAE mice. To explore the role of the enzymes, investigators have inhibited Arg-1 in EAE mice, during inductive and effector phases. Impairing Arg-1 altered immune response to myelin OD glycoprotein and improved EAE in mice. NO production is slightly increased, and there is less TNF-α synthesis. It is suggested that Arg-1 could regulate EAE through a NO-independent manner. An impaired immune response can be caused by inhibition of arginase. L-Ornithine, a metabolite from arginase pathway, is necessary for production of polyamine and proline, which control cell proliferation (Xu et al., 2003; Polis et al., 2020). The role of arginase is not clear; further studies are required to better understand the function of this enzyme in MS.

TGF-β suppresses inflammatory factors produced by microglia, thereby regulating the inflammatory response by these cells. Moreover, the critical role of this regulatory factor is to maintain tolerance *via* the regulation of lymphocyte proliferation, differentiation, and survival (Suzumura et al., 1993; Mirshafiey and Mohsenzadegan, 2009). TGF-β down-regulates the production of myelin toxic factors such as TNF-α and oxygen radicals and induces a switch of microglia from proinflammatory to become repair cells. A study demonstrates that in the cuprizone model the expression of several trophic factors is up-regulated in the course of DM and RM, namely, TGF-β and IGF-1 (Olah et al., 2012; Guerrero and Sicotte, 2020). This overexpression promotes OD differentiation and migration toward lesion site (Hinks and Franklin, 1999; Diemel et al., 2003; Traiffort et al., 2020). Furthermore, Hamaguchi et al. (2019) recently proposed that circulating TGF-β1 enters the CNS and could contribute to RM. The study provided evidence that TGF-β1 administration promotes RM by stimulating human OD maturation and restore neurological function in MS (Hamaguchi et al., 2019).

Researchers had also a growing interest in macrophage-colony stimulating factor (m-CSF) and its receptor in the past few years. Modulation of the CSF axis could be used as a therapeutic target for many diseases from cancer to neurodegenerative pathologies (Pons and Rivest, 2018). In the brain, microglia are affected by CSF1R activity depending on the context (Pons et al., 2020). Laflamme et al. (2018) have used two different methods to study the role of m-CSF in cuprizone-intoxicated mice. They observed a reduced number of OPCs in the corpus callosum of cuprizone-fed mice bearing a deletion in the CSF1R gene. Moreover, animals exhibited a reduction of the microglial inflammatory response and a higher level of myelin in areas of interest. These data indicate that impairing the m-CSF signaling pathway alters microglial phagocytosis, inflammatory activation, and OPC proliferation. In a second series of experiments, they administered m-CSF during the DM phase. Interestingly, inducing m-CSF signalization restrained myelin loss, boosted microglial activity, increased IGF-1 expression, and promoted the recruitment of OPCs at the lesion site. This suggests that m-CSF has a robust beneficial role in this model and promotes repair by conditioning microglia and OPCs (Laflamme et al., 2018). Despite the promising therapeutic potential, it remains unclear how m-CSF affects microglia. In this study, authors used a genetic construction specifically located in microglia to suppress the CSF1R gene. In line with these findings, another group that used an m-CSF-deficient mouse also provided evidence that m-CSF deficiency impairs RM process. The lack of this cytokine causes aberrant activation of astrocytes, which may contribute to axonal damages and disturbs RM (Wylot et al., 2019).

Another method to impair CSF1R signaling pathway is to use biochemical molecules, which interfere with a different part of the receptor. However, these molecules bring out different outcomes, depending on experimental settings. Tahmasebi et al. (2019) have shown amelioration of the general condition in cuprizone-fed mice following CSF1R blockade with PLX3397, a molecule that impairs CSF1R signaling. According to these results, a decrease in the microglial inflammatory response is observed in mice treated with PLX3397. The authors reported an increased rate of Olig-2 expression within the corpus callosum and showed that PLX3397 treatment could induce differentiation of OPCs. They hypothesized that the CSF1R inhibitor attenuates inflammation by microglia, and this could lead to a decrease in OPC death rate. These results are quite surprising because many studies have pointed out the beneficial role of inflammation in RM process and the strong positive action of CSF1R (Kerschensteiner et al., 1999; Arnett et al., 2003; Kotter et al., 2005; Olah et al., 2012; Miron et al., 2013; Rawji and Yong, 2013; Lampron et al., 2015; Luo et al., 2017; Laflamme et al., 2018). The major difference between chemical and genetic depletion of CSF1R is the specificity. Indeed, molecules are not or at least less specific than genetic ablation. It could have a cross-inhibitory reaction with other receptors from the same family, whereas genetic construction is restricted to a specific cell type. This may explain why results could be different between studies using molecular compounds to inhibit CSF1R.

## Conclusion

**Figure 1:**
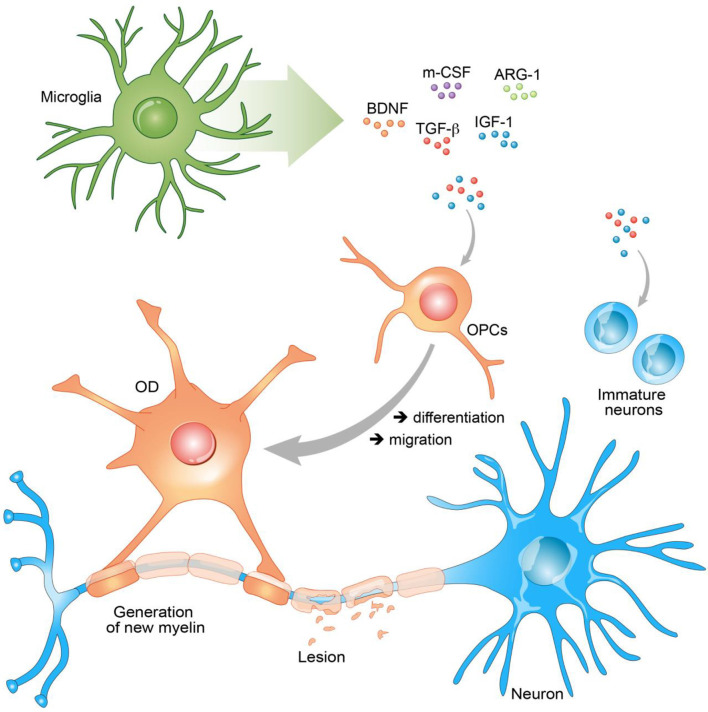
Secreted growth factors (GFs) promote remyelination (RM). Secreted GFs have the ability to stimulate neurons, oligodendrocyte progenitor cells (OPCs), and oligodendrocyte (OD) to promote RM. Brain neurotrophic–derived factor (BDNF), insulin growth factor 1 (IGF-1), arginase-1 (Arg-1), and transforming growth factor β (TGF-β) act on the differentiation of OPCs into ODs and their migration to the lesion site. In the same manner through the secretion of these GFs, microglia stimulate immature neurons and promote neurogenesis.

This review is an overview of the involvement of microglia in RM processes through production of GFs (Figure 1).

The role of microglia in MS is complex and requires extensive studies. However, the last decades provided a good insight on the implication of microglial in DM/RM processes. We have seen the importance of microglia reactivity in DM phase to set the conditions for RM. IGF-1, Arg-1, BDNF, TGF-β, and m-CSF have a beneficial effect on RM processes. Most of these molecules act on OPCs, while chronic inflammation induced by microglia leads to the death of ODs. As a central player, microglia are really attractive targets in neurodegenerative diseases, including Alzheimer disease and MS. Dysregulation of microglial function, activation, and secretion of GFs remains poorly understood. It is critical to fully comprehend the role and action of microglia to focus on new efficient therapies. The modulation of these GFs influences the differentiation and proliferation of OPCs. These results suggest that the modulation of microglia or OPCs in diseases may represent an interesting therapeutic strategy to promote RM.

## Author Contributions

VP and SR wrote the manuscript together.

## Conflict of Interest

The authors declare that the research was conducted in the absence of any commercial or financial relationships that could be construed as a potential conflict of interest.
